# Discontinuation of Denosumab and Associated Fracture Incidence: Analysis From the Fracture Reduction Evaluation of Denosumab in Osteoporosis Every 6 Months (FREEDOM) Trial

**DOI:** 10.1002/jbmr.1808

**Published:** 2013-03-18

**Authors:** Jacques P Brown, Christian Roux, Ove Törring, Pei-Ran Ho, Jens-Erik Beck Jensen, Nigel Gilchrist, Christopher Recknor, Matt Austin, Andrea Wang, Andreas Grauer, Rachel B Wagman

**Affiliations:** 1 Centre Hospitalier Universitaire de Québec–Centre Hospitalier de l'Université Laval (CHUQ-CHUL) Research CentreQuébec, Québec, Canada; 2 Paris Descartes University, Cochin Hospital, Assistance Publique–Hôpitaux de Paris (AP-HP)Paris, France; 3 Karolinska Institutet, SödersjukhusetStockholm, Sweden; 4 Amgen Inc.Thousand Oaks, CA, USA; 5 Hvidovre University HospitalHvidovre, Denmark; 6 Canterbury Geriatric Medical Research TrustChristchurch, New Zealand; 7 United Osteoporosis CentersGainesville, GA, USA

**Keywords:** DENOSUMAB, POSTMENOPAUSAL OSTEOPOROSIS, FRACTURE, DISCONTINUATION, OFF-TREATMENT

## Abstract

Osteoporosis is a chronic disease and requires long-term treatment with pharmacologic therapy to ensure sustained antifracture benefit. Denosumab reduced the risk for new vertebral, nonvertebral, and hip fractures over 36 months in the Fracture Reduction Evaluation of Denosumab in Osteoporosis Every 6 Months (FREEDOM) trial. Whereas discontinuation of denosumab has been associated with transient increases in bone remodeling and declines in bone mineral density (BMD), the effect on fracture risk during treatment cessation is not as well characterized. To understand the fracture incidence between treatment groups after cessation of investigational product, we evaluated subjects in FREEDOM who discontinued treatment after receiving two to five doses of denosumab or placebo, and continued study participation for ≥7 months. The off-treatment observation period for each individual subject began 7 months after the last dose and lasted until the end of the study. This subgroup of 797 subjects (470 placebo, 327 denosumab), who were evaluable during the off-treatment period, showed similar baseline characteristics for age, prevalent fracture, and lumbar spine and total hip BMD *T*-scores. During treatment, more placebo-treated subjects as compared with denosumab-treated subjects sustained a fracture and had significant decreases in BMD. During the off-treatment period (median 0.8 years per subject), 42% versus 28% of placebo- and denosumab-treated subjects, respectively, initiated other therapy. Following discontinuation, similar percentages of subjects in both groups sustained a new fracture (9% placebo, 7% denosumab), resulting in a fracture rate per 100 subject-years of 13.5 for placebo and 9.7 for denosumab (hazard ratio [HR] 0.82; 95% confidence interval [CI], 0.49–1.38), adjusted for age and total hip BMD *T*-score at baseline. There was no apparent difference in fracture occurrence pattern between the groups during the off-treatment period. In summary, there does not appear to be an excess in fracture risk after treatment cessation with denosumab compared with placebo during the off-treatment period for up to 24 months. © 2013 American Society for Bone and Mineral Research.

## Introduction

Sustained benefit of a therapeutic agent for a chronic condition generally requires continued treatment. Effects of therapeutic agents often are not sustained once treatment is discontinued, including in chronic diseases, such as hypertension and diabetes mellitus. Among osteoporosis therapies, reversibility of treatment effect has been observed with some, but not all, pharmacologic interventions, as judged by bone mineral density (BMD) and biochemical markers of bone turnover, or bone turnover markers (BTMs), but effect on fracture risk is less clear. Postmenopausal estrogen therapy and estrogen receptor agonists/antagonists have a similar pattern of reversibility with therapy discontinuation as assessed by BMD, BTMs, or both.^1^–^12^ Large observational studies with hormone therapy withdrawal have not shown excess in osteoporotic fracture risk after therapy discontinuation, and data supporting hip fracture incidence are inconclusive.^13^–^16^ Reversibility, as measured by BMD and BTMs, also has been observed with the anabolic agent, teriparatide.^17^, ^18^

Evidence of reversibility within the bisphosphonate class varies by compound affinity to hydroxyapatite: etidronate, risedronate, and ibandronate have lower affinity, and alendronate and zoledronic acid have higher affinity.^19^–^21^ Clinical consequences of adsorption affinity are reflected when treatment with bisphosphonate is discontinued. Gradual increases in BTMs and declines in BMD have been observed 12 months after discontinuation of risedronate^22^ compared with gradual changes over a few years after discontinuation of alendronate or zoledronic acid.^23^, ^24^ Discontinuation may affect bone turnover in trabecular and cortical compartments and microarchitecture, which are important for bone strength and fracture risk. Fracture outcomes, however, are less well-documented due to insufficient sample sizes during the follow-up period.^22^–^24^

Denosumab is a fully human monoclonal antibody (immunoglobulin G subclass 2 [IgG2]) with high affinity and specificity for human receptor activator of NF-κB ligand (RANKL), and neutralizes the activity of human RANKL, similar to the action of endogenous osteoprotegerin. In blocking RANKL, denosumab inhibits osteoclast formation, function, and survival, thereby decreasing bone resorption and increasing bone mass and strength in both trabecular and cortical bone. The 36-month data from the randomized, double-blind, placebo-controlled, phase 3 Fracture Reduction Evaluation of Denosumab in Osteoporosis Every 6 Months (FREEDOM) trial in women with postmenopausal osteoporosis demonstrated that denosumab treatment reduced the incidence of new vertebral fractures, nonvertebral fractures, and hip fractures when compared with placebo.^25^

As a soluble inhibitor of RANKL, denosumab does not incorporate into the bone matrix, and therefore, its effects are reversible with therapy discontinuation, as measured by BMD, BTMs, and bone histomorphometry.^26^–^28^ Because none of the studies in the denosumab clinical trial program was designed to appropriately assess differences in fracture rates after treatment discontinuation, implications for fracture risk are not well characterized. To understand the fracture incidence between treatment groups after cessation of investigational product (IP), we retrospectively evaluated subjects in FREEDOM who discontinued denosumab or placebo and had sufficient follow-up time on study to assess clinical outcomes.

## Methods

### Study population

Subjects included in this analysis were enrolled in the international, multicenter, randomized, double-blind, placebo-controlled pivotal phase 3 fracture trial (FREEDOM), which has been previously reported.^25^ Briefly, postmenopausal women aged 60 to 90 years (*N* = 7808) with a BMD *T*-score of < −2.5 at either the lumbar spine or total hip and ≥−4.0 at both sites were randomly assigned to receive subcutaneous injections of placebo or 60 mg denosumab every 6 months for 36 months. All subjects received daily calcium (≥1 g) and vitamin D (≥400 IU) supplementation. Women with > 2 moderate vertebral fractures or any severe vertebral fracture were excluded. Subjects who had used oral bisphosphonates for >36 months cumulatively were excluded; subjects with ≤36 months of oral bisphosphonate use and no use 12 months prior to enrollment were eligible. The study was conducted in accordance with Good Clinical Practice and the Declaration of Helsinki. Local institutional review board approval was obtained for the protocol and all subjects provided informed consent prior to any study-related procedures.

### Subject selection for treatment discontinuation analysis

**Fig. 1:**
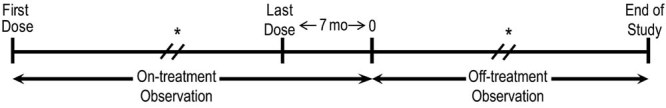
On-treatment and off-treatment observation periods. *Indicates not drawn to scale. Duration of on-treatment and off-treatment observation periods varies from subject to subject. mo = month.

In this retrospective analysis, we evaluated subjects in FREEDOM who discontinued treatment after receiving two to five doses of IP, either placebo or denosumab, and continued study participation for ≥7 months (≥6 months since the last dose + 1-month study visit window). A minimum of the first two doses was required because the earliest time point at which antifracture efficacy has been observed with denosumab treatment was at 12 months^25^ and therefore, the maximum off-treatment observation period for this group could have been 24 months. Subjects who had received up to five doses were included in the analysis as this permitted the minimum off-treatment observation period to begin ≥7 months after the last dose of IP (Fig. 1).

The reasons for IP discontinuation included ineligibility determined, protocol deviation, noncompliance, adverse event, consent withdrawn, subject request, disease progression, requirement for other therapy, administrative decision, lost to follow-up, death, or other. If a subject developed an on-study fracture, the participant had the opportunity to discontinue study IP and remain enrolled to continue with study assessments. Similarly, subjects who developed significant BMD reduction, defined as >7% BMD reduction at the total hip within any 12-month period, ≥10% BMD reduction at the total hip from baseline at any time point, or total hip BMD *T*-score < −4 at any time point, may have discontinued IP but remained enrolled.

### Statistical methods

Subject demographics at FREEDOM entry baseline, on-study vertebral and nonvertebral fracture events excluding skull, face, mandible, metacarpals, fingers, and toes, and reasons for IP discontinuation were summarized descriptively. Multiple nonvertebral fractures that occurred on the same day for an individual subject were treated as a single event. The fractures were analyzed as recurrent events using the Andersen–Gill formulation of the Cox proportional hazards model adjusting for baseline age and total hip BMD *T*-score.^29^ The hazard ratio (HR) between the treatment groups (denosumab versus placebo) during the off-treatment period and the 95% confidence intervals (CI) were calculated using the robust sandwich variance estimate. Time to first osteoporosis-related fracture during the off-treatment period for both treatment groups was depicted using the Kaplan-Meier method.

## Results

### Baseline demographics

Of the 1783 subjects who discontinued IP in FREEDOM, 797 subjects (470 placebo, 327 denosumab) were included in the off-treatment analysis. Placebo- and denosumab-treated subjects included in the off-treatment analysis showed similar baseline characteristics at FREEDOM study entry with respect to age, prevalent fracture, and lumbar spine and total hip BMD *T*-scores as subjects who had discontinued IP but did not meet criteria for this assessment of fracture risk (Table 1). The mean follow-up time per subject during the off-treatment period (from last dose + 7 months to end of study) was 0.8 years for both treatment groups. Baseline characteristics of the subjects included in the off-treatment analysis were consistent with those of the overall FREEDOM cohort.

**Table 1:** Baseline Characteristics: Subjects Who Discontinued Treatment and Subjects in the FREEDOM Study

	Off-treatment analysis					
			
	Included		Excluded		FREEDOM^25^	
			
	Placebo (*N*1 = 470)	Denosumab (*N*1 = 327)	Placebo (*N*2 = 520)	Denosumab (*N*2 = 466)	Placebo (*N*3 = 3906)	Denosumab (*N*3 = 3902)
Baseline characteristics						
Age, years (mean ± SD)	73 ± 5	73 ± 5	74 ± 5	73 ± 5	72 ± 5	72 ± 5
Prevalent fracture, *n* (%)						
Vertebral	122 (26)	89 (27)	155 (30)	107 (23)	915 (23)	929 (24)
Nonvertebral	149 (32)	107 (33)	168 (32)	149 (32)	1177 (30)	1163 (30)
BMD *T*-score (mean ± SD)						
Lumbar spine	−2.8 ± 0.7	−2.8 ± 0.8	−2.8 ± 0.7	−2.8 ± 0.7	−2.8 ± 0.7	−2.8 ± 0.7
Total hip	−2.1 ± 0.9	−2.0 ± 0.9	−2.1 ± 0.8	−2.0 ± 0.8	−1.9 ± 0.8	−1.9 ± 0.8
Prior bisphosphonate use, *n* (%)	62 (13)	46 (14)	66 (13)	61 (13)	521 (13)	472 (12)
Duration, years						
*n*1	54	41	56	54	460	415
Mean	1.0	1.0	0.9	0.9	0.9	0.9
Median (Q1, Q3)	0.8 (0.3, 1.4)	1.0 (0.1, 2.0)	0.5 (0.2, 1.0)	1.0 (0.2, 1.5)	0.5 (0.2, 1.6)	0.8 (0.2, 1.3)
Per-subject follow-up, years^a^						
Mean	0.8	0.8	0.3	0.3	2.8	2.8
Median (Q1, Q3)	0.5 (0.3, 1.4)	0.5 (0.2, 1.4)	0 (0,0)	0 (0, 0)	3.0 (3.0, 3.0)	3.0 (3.0, 3.0)

*N*1 = number of subjects who discontinued treatment after receiving two to five doses of IP, either placebo or denosumab, and continued study participation for ≥7 months after the last dose.

*N*2 = number of subjects who discontinued treatment after receiving only one dose of IP, either placebo or denosumab, or subjects who discontinued treatment but remained on study for <7 months after the last dose.

*N*3 = total number of randomized subjects.

*n*1 = number of subjects with available data for bisphosphonate duration.

FREEDOM = Fracture Reduction Evaluation of Denosumab in Osteoporosis Every 6 Months; BMD = bone mineral density; Q1 = quartile 1; Q3 = quartile 3; IP = investigational product.

a From last dose + 7 months to end of study.

### On-treatment experience during FREEDOM

During the on-treatment period in FREEDOM, more subjects treated with placebo as compared with denosumab sustained a fracture (19% for placebo versus 11% for denosumab) and had significant decreases in BMD (17% for placebo versus 1% for denosumab) (Table 2). Reasons for study discontinuation for both treatment groups are also shown in Table 2. There was a higher number of denosumab-treated subjects who discontinued due to malignant neoplasm, specifically breast cancer; overall, malignancies related to the breast were 0.7% for placebo versus 0.9% for denosumab.^30^, ^31^ During the off-treatment period, 42% of placebo-treated subjects versus 28% of denosumab-treated subjects initiated other therapy after the last dose. Table 3 provides a summary of the other therapies initiated. More placebo-treated subjects started bisphosphonate or parathyroid hormone (PTH) therapy than denosumab-treated subjects. More denosumab-treated subjects initiated antineoplastic therapy and aromatase inhibitor therapy, which is consistent with the difference in the discontinuation rate due to malignant neoplasm observed in the denosumab-treated group (Table 3).

**Table 2:** Subject Characteristics at Treatment Discontinuation

	Placebo (*N* = 470) *n* (%)	Denosumab (*N* = 327) *n* (%)
Number of doses received		
2	114 (24)	86 (26)
3	138 (29)	99 (30)
4	90 (19)	68 (21)
5	128 (27)	74 (23)
Fracture during treatment^a^	90 (19)	36 (11)
Significant BMD reduction^b^ during treatment^a^	80 (17)	4 (1)
Treatment discontinuation as a result of:		
Adverse event	124 (26)	104 (32)
Malignancy	20 (4)	29 (9)
Consent withdrawn	92 (20)	64 (20)
Requirement for other therapy^c^	60 (13)	24 (7)
Disease progression	56 (12)	5 (2)
Subject request	54 (12)	54 (17)
Other	17 (4)	22 (7)
Lost to follow-up	16 (3)	12 (4)
Protocol deviation	16 (3)	11 (3)
Death	8 (2)	2 (1)
Noncompliance	4 (1)	6 (2)
Administrative decision	3 (1)	7 (2)
Started other therapy after last dose	197 (42)	90 (28)

*N* = number of subjects who discontinued treatment after receiving two to five doses of IP, either placebo or denosumab, and continued study participation for ≥7 months after the last dose.

BMD = bone mineral density; IP = investigational product.

a Treatment period = first dose through last dose + 7 months.

b Significant BMD reduction is defined as >7% BMD reduction at the total hip within any 12-month period, ≥10% BMD reduction at the total hip from baseline at any time point, or total hip BMD *T*-score < −4 at any time point.

c As determined by the discretion of the investigator.

**Table 3:** Other Therapies Initiated After the Last Dose of IP in Subjects Who Were Included in the Off-Treatment Analysis

	Placebo (*N* = 470) *n* (%)	Denosumab (*N* = 327) *n* (%)
Started other therapy after last dose^a^	197 (42)	90 (28)
Bisphosphonates	176 (90)	70 (78)
SERMs	10 (5)	5 (6)
Hormone therapies^b^	4 (2)	2 (2)
PTH or a derivative	7 (4)	0 (0)
Strontium ranelate	6 (3)	3 (3)
Calcitonin	1 (1)	1 (1)
Systemic corticosteroids^c^	38 (19)	17 (19)
Antineoplastics	10 (5)	12 (13)
Aromatase inhibitors	3 (2)	5 (6)

*N* = number of subjects who discontinued treatment after receiving two to five doses of IP, either placebo or denosumab, and continued study participation for ≥7 months after the last dose.

IP = investigational product; SERM = selective estrogen receptor modulator; PTH = parathyroid hormone.

a Percentages are based on *N*; the remaining percentages are based on the number of subjects who started other therapy after last dose.

b May include estrogen alone, or estrogen and progestin.

c Includes those subjects exposed to systemic corticosteroids >5 mg/day for >3 months (no need to be consecutive).

### Treatment discontinuation experience during FREEDOM

**Fig. 2:**
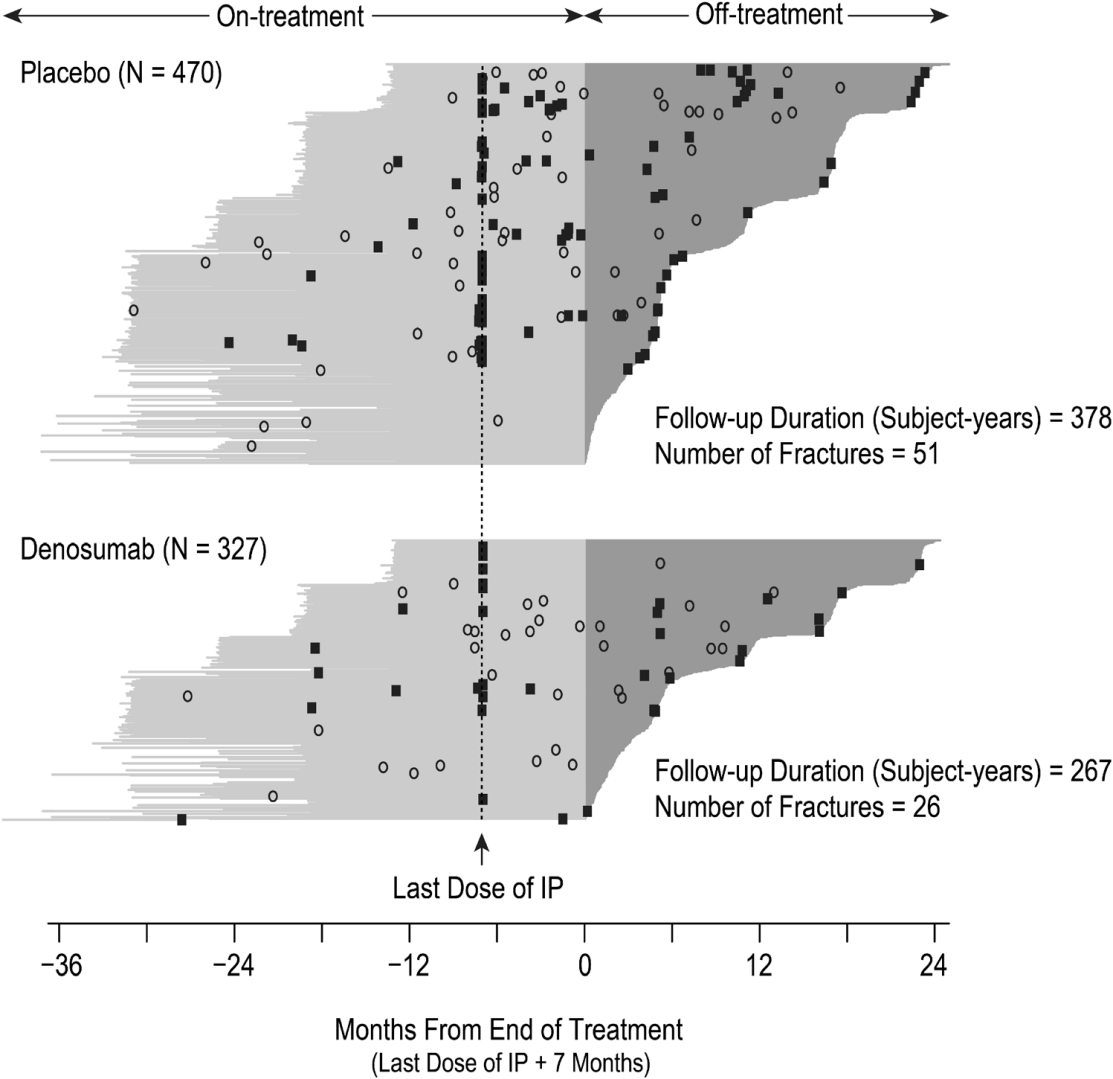
Each horizontal line represents the length of study duration for each subject included in the analysis. The vertical dashed line represents the last dose of IP and the zero time point denotes end of the on-treatment period or beginning of off-treatment period. Light gray shading represents the on-treatment period and dark gray shading represents the off-treatment period. The subjects are arranged by decreasing off-treatment duration. Vertebral fractures are depicted as closed squares and nonvertebral fractures as open circles.

Individual subject fracture occurrences for vertebral and nonvertebral fractures with respect to on- and off-treatment duration are depicted in Fig. 2. There was no apparent difference in fracture occurrence pattern between the groups during both the on- and off-treatment periods as represented by the apparently random occurrence of events with respect to time. Vertebral fracture clustering was observed at the last dose of IP and corresponded to the annually scheduled lateral spine X-ray assessments, whereas nonvertebral fractures were collected on an ongoing basis as they occurred.^25^

Similar percentages of subjects in both groups sustained an osteoporosis-related fracture during this follow-up period (9% placebo, 7% denosumab). With 470 placebo-treated subjects followed for a total of 378 subject-years and 327 denosumab-treated subjects followed for a total of 267 subject-years, the overall fracture rate per 100 subject-years was 13.5 for placebo and 9.7 for denosumab (HR 0.82; 95% CI, 0.49–1.38), adjusted for age and total hip BMD *T*-score at baseline (Table 4). The fracture HR between treatment groups remained consistent after the removal of subjects who initiated an effective osteoporosis therapy (eg, bisphosphonates, selective estrogen receptor modulators [SERMs], PTH) after the last dose of IP (HR 0.85; 95% CI, 0.40–1.79).

**Table 4:** Osteoporotic Fracture Occurrences During Off-Treatment

	Placebo (*N* = 470)	Denosumab (*N* = 327)
Subjects with fracture, *n* (%)	43 (9)	23 (7)
Follow-up duration (subject-years)	378	267
Number of fractures	51	26
Vertebral	35	15
Nonvertebral	16	11
Hip	2	1
Fracture rate per 100 subject-years	13.5	9.7
Vertebral	9.3	5.6
Nonvertebral	4.2	4.1
Hip	0.5	0.4

*N* = number of subjects who discontinued treatment after receiving two to five doses of IP, either placebo or denosumab, and continued study participation for ≥7 months after the last dose.

IP = investigational product.

**Fig. 3:**
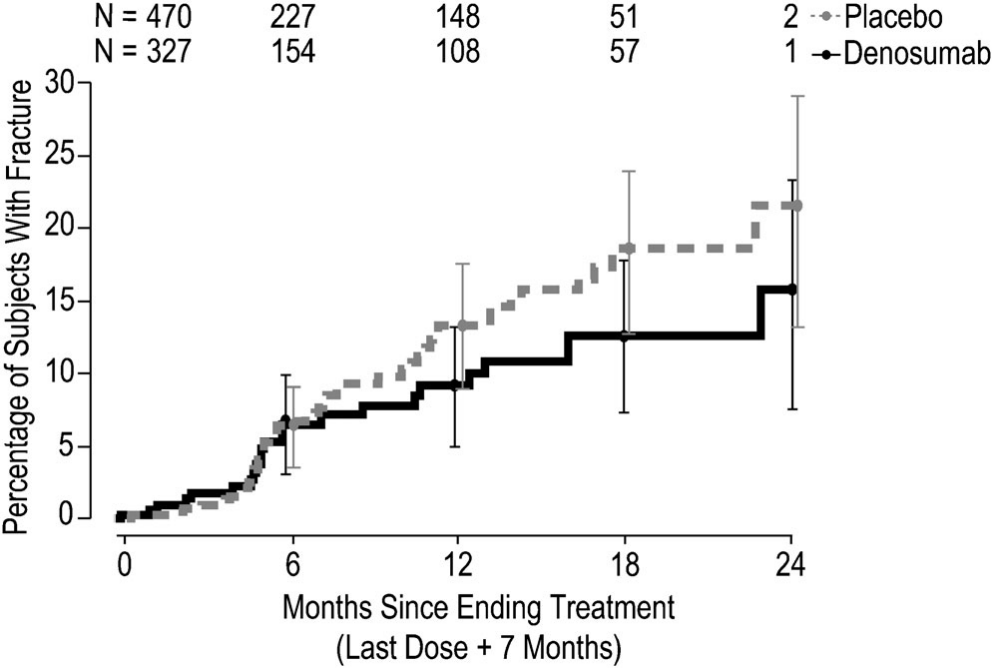
Error bars represent 95% confidence intervals. *N* = subjects at risk for fracture of subjects who discontinued treatment after receiving two to five doses of IP, either placebo or denosumab, and continued study participation for ≥7 months after the last dose.

Time to first osteoporotic fracture during the off-treatment period is shown in Fig. 3. The fracture rates were similar between treatment groups during the first 6 months in the off-treatment period. A higher fracture rate for the placebo group was observed after the initial 6 months but was not statistically different from the denosumab group throughout the off-treatment observational period.

## Discussion

The effects of denosumab on BMD and bone remodeling are reversible after treatment cessation due to its mechanism of action and lack of incorporation into the bone matrix, as we previously have shown.^26^–^28^ Denosumab discontinuation has been associated with increases in BTMs above baseline, which transiently increased above the premenopausal reference range and approached pretreatment levels by 18 to 24 months after therapy cessation.^26^ BMD generally returned to pretreatment levels at all measured sites (but remained above levels in the placebo group), indicating that the magnitude of the reduction in BMD following discontinuation of denosumab treatment was similar to the level of increase in BMD during treatment.^26^ These changes in bone turnover and BMD are internally consistent as transient increases in remodeling are associated with declines in bone density. The current study investigated whether this increased bone remodeling had an effect on fracture risk in postmenopausal women with osteoporosis who discontinued treatment, either placebo or denosumab, in the 36-month FREEDOM study. For those subjects included in this analysis, similar percentages of subjects in both groups sustained an osteoporosis-related fracture during the follow-up period (9% placebo, 7% denosumab), suggesting that the previously reported transient increases in bone remodeling and declines in BMD upon denosumab discontinuation were not associated with excess fracture risk for up to 24 months. Since more placebo-treated subjects sustained a fracture and had significant BMD decreases during the on-treatment period, a higher fracture incidence than denosumab-treated subjects may be expected during the off-treatment period. More placebo-treated subjects initiated other osteoporosis therapies, specifically a bisphosphonate, during the off-treatment period, which would have been expected to lower their fracture rate. Interestingly, the fracture incidence observed in the placebo group remained higher compared with the denosumab group.

Fracture data during the off-treatment period have been more difficult to accrue, in part due to the ethics of discontinuing osteoporosis treatment in an individual at increased risk for fracture. The current analysis was undertaken to help address the effects of denosumab treatment cessation on fracture risk. Other available information includes a study of postmenopausal women with low bone mass who discontinued denosumab treatment for 24 months, which collected the most complete off-treatment fracture data in a radiographic evaluation of the spine that was performed at 48 months in all subjects and evaluated by a central reader to confirm vertebral fractures. In addition, the central reader evaluated X-rays to confirm fractures in subjects who reported an adverse event of fracture. The incidence of osteoporosis-related fractures was overall balanced between groups.^26^

Although this analysis addresses important questions, there are some limitations in the approach: the evaluation was post hoc, there was a relatively limited follow-up observation period of fractures (median follow-up of 0.8 years per subject), and subjects who remained on study <7 months were excluded.

In summary, we conclude from this analysis in postmenopausal women with osteoporosis that there does not appear to be an excess in fracture risk associated with denosumab treatment cessation. As would be expected with a reversible agent, the beneficial effect of denosumab treatment on fracture risk reduction is not sustained once therapy is discontinued. To ensure long-term benefit for a chronic condition, such as osteoporosis, continued treatment is essential for high-risk patients.

## Disclosures

JPB has received research grants from Abbott, Amgen Inc., Bristol-Myers Squibb, Eli Lilly, Merck, Novartis, Pfizer, Roche, sanofi-aventis, Servier, Takeda, and Warner Chilcott; has received consulting fees or other remuneration from Amgen, Inc., Eli Lilly, Merck, Novartis, sanofi-aventis, and Warner Chilcott; and has served on the speaker's bureau for Amgen, Inc., Eli Lilly, and Novartis. CR has received research grants from Novartis, Amgen Inc., Bongrain, and MSD; and has received consulting fees or other remuneration from Novartis, Amgen Inc., MSD, Roche, Servier, and Lilly. OT has served as a consultant or advisor or has been on the speaker's bureau for Takeda-Nycomed, Amgen Inc., GSK, LuBio, and Eli Lilly. JEBJ has served as a consultant or advisor or has been on the speaker's bureau for Eli Lilly, Takeda, Nycomed, Amgen Inc., Novartis, and MSD. NG has received consulting fees or other remuneration from Amgen Inc., Eli Lilly, MSD, and Novartis. CR has received consulting fees or other remuneration from Zelos, Takeda, Dramatic Health, Novartis, and Eli Lilly; and has received lecture fees from Amgen Inc., Novartis, and Publicis Meetings. PRH, MA, AW, AG, and RBW are employees of Amgen Inc. and own stock/stock options in Amgen Inc.
